# Invasive therapies for Parkinson’s disease: an adapted excerpt from the guidelines of the German Society of Neurology

**DOI:** 10.1007/s00415-025-12915-6

**Published:** 2025-02-22

**Authors:** René Reese, Thomas Koeglsperger, Christoph Schrader, Lars Tönges, Günther Deuschl, Andrea A. Kühn, Paul Krack, Alfons Schnitzler, Alexander Storch, Claudia Trenkwalder, Günter U. Höglinger, for the German Parkinson‘s Guideline Group

**Affiliations:** 1 Department of Neurology, Rostock University Medical Center, Rostock, Germany; 2 Department of Neurology, LMU University Hospital, LMU Munich, Munich, Germany; 3 Department of Neurology, Hannover Medical School, Hannover, Germany; 4 Department of Neurology, St. Josef-Hospital, Ruhr University Bochum, Bochum, Germany; 5 Neurodegeneration Research, Protein Research Unit Ruhr (PURE), Ruhr University Bochum, Bochum, Germany; 6 Department of Neurology, University Hospital Schleswig-Holstein, Kiel, Germany; 7 Movement Disorder and Neuromodulation Unit, Department of Neurology, Charité, University Medicine Berlin, Berlin, Germany; 8 Movement Disorders Center, Department of Neurology, University Hospital (Inselspital) and University of Bern, Bern, Switzerland; 9 Institute of Clinical Neuroscience and Medical Psychology, Medical Faculty, Heinrich-Heine University, Düsseldorf, Germany; 10 Department of Neurology, Medical Faculty, Heinrich-Heine University, Düsseldorf, Germany; 11 Center for Transdisciplinary Neurosciences Rostock (CTNR), Rostock University Medical Center, Rostock, Germany; 12 German Center for Neurodegenerative Diseases (DZNE) Rostock/Greifswald, Rostock, Germany; 13 Paracelsus-Elena-Klinik, Kassel, Germany; 14 Department of Neurosurgery, University Medical Center Göttingen, Göttingen, Germany; 15 German Center for Neurodegenerative Diseases (DZNE), Munich, Germany; 16 Munich Cluster for Systems Neurology (SyNergy), Munich, Germany; 17 Klinik und Poliklinik für Neurologie, Universitätsmedizin Rostock, Gehlsheimer Str. 20, 18147 Rostock, Germany

**Keywords:** Parkinson’s disease, Invasive therapies, Deep brain stimulation (DBS), Pump therapy, Ablation

## Abstract

**Background:**

Parkinson’s disease (PD) is characterized by hypokinetic motor symptoms, tremor, and various non-motor symptoms with frequent fluctuations of symptoms in advanced disease stages. Invasive therapies, such as deep brain stimulation (DBS), ablative therapies, and continuous subcutaneous or intrajejunal delivery of dopaminergic drugs via pump therapies are available for the management of this complex motor symptomatology and may also impact non-motor symptoms. The recent update of the clinical guideline on PD by the German Neurological Society (Deutsche Gesellschaft für Neurologie e.V.; DGN) offers clear guidance on the indications and applications of these treatment options.

**Methods:**

The guideline committee formulated diagnostic questions for invasive therapies and structured them according to the PICOS framework (Population–Intervention–Comparisons–Outcome–Studies). A systematic literature review was conducted. Questions were addressed using the findings from the literature review and consented by the guideline committee.

**Results:**

Specific recommendations are given regarding (i) the optimal timing for starting invasive therapies, (ii) the application of DBS, (iii) the use of pump therapies in advanced PD, (iv) the indications for ablative procedures, and (iv) selecting the most appropriate therapy according to individual patient characteristics.

**Conclusion:**

This review is an adapted excerpt of the chapters on the use of invasive therapies in PD of the novel German guideline on PD. Clear recommendations on the use of treatment options for advanced PD are provided.

## Introduction

Parkinson’s disease (PD) is a progressive neurodegenerative disorder characterized by both long-term clinical deterioration and short-term fluctuations in motor (motor fluctuations; MF) and non-motor symptoms (non-motor fluctuations; NMF) throughout day and night [1]. Tremor is also a common symptom of PD and may even be clinically and functionally disabling. It may manifest as resting and postural tremor of the extremities but can affect any other body part. MF, both hypokinetic and hyperkinetic, relate to variable effectiveness of dopaminergic medication with unpredictable changes in symptom severity and overall unsatisfactory symptom control [2]. These fluctuations affect approximately 80% of patients after a decade [3]. The most common hypokinetic fluctuations in PD are early morning Off, where symptoms reappear due to insufficient nighttime dopamine replacement, and wearing Off, which involves re-emergence of motor and non-motor symptoms before the next medication dose. Hyperkinetic fluctuations are characterized by uncontrolled movements or muscle contractions, such as dyskinesia or dystonia, often linked to dopaminergic drug intake in the disease’s advanced phases [4]. MF substantially affect quality of life (QoL), activities of daily living, cognition, stigma and bodily discomfort which underscores the importance of their prevention, delaying of onset and clinical management [5, 6]. Beyond the core motor symptoms, NMF affect 60% to 97% of PD patients and can fluctuate similarly to MF [7] [8]. These symptoms encompass neuropsychiatric, dysautonomic, and sensory manifestations, including pain, significantly impacting patients’ QoL and sometimes surpassing the influence of MF [9–11].

MF, some non-MF, and tremor can be treated by several advanced invasive therapies once oral medication provides insufficient symptom control. Invasive therapies for PD include deep brain stimulation (DBS) of the subthalamic nucleus (STN), globus pallidus internus (GPi), and ventral intermediate nucleus (VIM) of the thalamus, continuous subcutaneous or intrajejunal delivery of dopaminergic drugs via pump therapies, radiofrequency thermocoagulation or MR-guided focused ultrasound (MRgFUS). The indications for these therapies vary depending on age, the clinical symptomatology, the severity of fluctuations, and further individual patient characteristics. DBS involves implanting electrodes in specific brain areas to modulate neural activity, typically recommended for patients with advanced PD experiencing significant MFs or dyskinesias or tremor despite optimal medical therapy. Alternatively, pump therapies are suitable for patients who require continuous drug delivery to manage severe motor symptoms but may be less effective in controlling tremor. Ablative procedures, in particular unilateral MRgFUS, which target and destroy specific brain regions that are implicated in PD symptoms, may especially be considered in PD tremor with asymmetrical severity if DBS is not suitable.

This review is an English translation of the chapters on the use of invasive therapies in PD of the novel German guideline on Parkinson’s disease (PD) [12]. For reasons of clarity and readability, the original guideline text was adapted, bundled, or restructured for the current review if necessary. Moreover, some very recent topics on invasive therapies were added due to the time gap between literature search for the German guidelines and this current article. The PICO questions and the summarizing recommendations including the degree of agreement from the experts’ consensus conferences stated at the end of each chapter in this article were exactly translated from the German guideline. The authors of this article were the authors of the chapters on invasive therapies of the German guideline. Further information and references can be found in the original document https://register.awmf.org/de/leitlinien/detail/030-010.

## Methods

The recommendations and degree of consensus stated in the current German PD Guideline [12] adhere to the standard criteria for guidelines of level S2k of the German Working Group of the Scientific Medical Societies (Arbeitsgemeinschaft der Wissenschaftlichen Medizinischen Fachgesellschaften e.V., AWMS, https://register.awmf.org/de/start). The guideline was developed by the German Neurological Society (Deutsche Gesellschaft für Neurologie e.V., DGN), with G.H. and C.T. serving as the coordinating authors (steering group). The chapter authors of the guideline were selected by the steering group based on their clinical and scientific expertise (expert group). The steering group formulated key questions derived from national and international guidelines for Parkinson’s disease, namely NICE (www.nice.org.uk), AWMF (www.awmf.org), and the European Academy of Neurology (www.ean.org). The steering and expert groups supplemented the initial set of key questions if necessary to ensure full coverage of the topics. The following PICO criteria were used as a framework to formulate the literature search strategies to ensure comprehensive searches:

P (Population): e.g., adults (> 18 years) with (suspected) PD and/or (if applicable) atypical/secondary Parkinsonian disorders and/or (if applicable) essential tremor.

I (Intervention): e.g., deep brain stimulation.

C (Comparison): e.g., clinical diagnosis of PD established by movement disorder specialists based on international consensus criteria (established/confirmed at follow-up visits).

O (Outcomes): e.g., improvement of PD motor symptoms.

S (Studies): e.g., original articles (including observational studies, randomized control trials), systematic reviews, meta-analyses, and case series.

A literature search was conducted in the PubMed database (https://pubmed.ncbi.nlm.nih.gov/) from January 2016 to December 2021 and included publications in German or English. The restriction to this database was decided by the paucity and insignificance of references obtained from other databases during the previous guideline’s literature search. Literature published prior to 2016 was retrieved from the former guideline version [15]. The literature was made available to the expert group to prepare the guideline chapters, namely background texts and summarizing recommendations according to the respective key questions. Lead authors of all chapters and invited representatives of participating specialist societies then voted in a Delphi process on these summarizing recommendations. The recommendations could already be adopted if more than 95% of the votes were Yes. The steering group reviewed these votes and comments forwarding them to the respective chapter authors who then prepared revisions of the recommendations where needed. The revised recommendations were presented, discussed, and adopted if necessary in a series of five online consensus conferences again with the lead authors of all chapters and the invited representatives of participating specialist societies. Following the National Institute of Health (NIH) guidelines and AWMF specifications, the degree of recommendations were expressed as should, might, or can. A strong consensus was noted with > 95% of agreement, a consensus with > 75–95%, a majority agreement with > 50–75%, and no majority agreement with < 50% of those eligible to vote.

## Results

### Appropriate time points for invasive therapies

***Rationale****:* In the early stages of PD, symptoms are often manageable with oral treatments. However, as the disease progresses to later stages, additional and sometimes invasive treatment options are required. This chapter aims to explore the clinical scenarios that should prompt the evaluation of device-assisted therapies (DATs).

***Background:*** Principal treatment options encompass a spectrum of pharmacological and interventional strategies. Initial steps typically involve optimizing oral medications such as levodopa, with adjustments in dosage, frequency, and soluble formulation to manage unpredictable MFs throughout the day. Long-acting dopamine agonists, on-demand subcutaneous apomorphine injections, and enzyme inhibitors to delay levodopa degradation are among further options to treat MF in advanced PD. When conventional treatments fail to provide sufficient control, invasive approaches should be considered. Tools like MANAGE-PD, MAF/D, and CEDEPA are screening and decision-making instruments designed to assist healthcare providers in managing PD symptoms. These tools may help to identify PD patients not adequately controlled with oral medication. These tools assess various symptoms, including motor fluctuations and functional impairments, to determine whether a patient could benefit from DAT, such as pump therapies or DBS [13–15].

***Evidence base*:** The recommendation is based on two consensus papers and a cohort study where treatment criteria were retrospectively evaluated in clinical practice [16–18].

***Results****:* Invasive therapies should be considered when medication adjustments prove insufficient. Patients should be informed about these options early in the disease course, once MFs or tremor become clinically significant. Expert recommendations from programs like NAVIGATE PD suggest specific criteria for discussing invasive therapies, emphasizing impaired QoL despite optimized medical treatment. Patients should be considered candidates for invasive therapies and referred to a specialist for these procedures when levodopa is required more than five times a day (with intake intervals of less than 3 h) or patients experience more than 2 h of Off phases or more than 1 h of (troublesome) dyskinesia during the day despite optimized oral, sublingual, inhalative or transdermal treatment. Indicators such as painful dystonia, Off freezing, and levodopa-dependent NMFs should also be considered. Invasive therapeutic interventions should also be considered in cases where a clinically and functionally relevant tremor is present. Symptom severity and impact on QoL should as well guide decision-making, prompting referral to movement disorders specialists regardless of disease duration [16, 17].

**Table:** 

Based on the available evidence, the following recommendations were agreed upon in the German PD Guideline Patients with PD should be informed about invasive treatments once first MFs occur In patients with PD fulfilling at least one of the following criteria, the indication for an invasive procedure should be discussed: ≥ 5 intake times of levodopa /day (corresponding to intake intervals of < 3 h) ≥ 2 h Off symptoms/day ≥ 1 h troublesome dyskinesia/day Activities of daily living (ADL) and QoL (as measured by e.g., the PDQ-39) scores should regularly be included in the decision for or against an invasive therapy Adequate treatment episodes with levodopa in combination with a dopamine agonist, MAO-B and COMT inhibitor should have been ineffective prior to indicating for invasive therapies These criteria are neither necessary nor sufficient for the indication but may provide guidance	
**Consensus strength:**	**95.2%, strong consensus**

### Therapeutic options

#### Deep brain stimulation

***Comparative effectiveness and safety of DBS (STN, GPi, VIM) vs. standard oral/transdermal therapy in PD management with MFs, with and without dyskinesias***

***Rationale:*** This section evaluates the safety and effectiveness of DBS (STN, GPi, VIM) compared to standard therapies, focusing on outcomes in patients with and without dyskinesias, to guide treatment decisions in advanced PD.

***Evidence base:*** Large randomized and controlled studies exist only for the comparison of STN-DBS with oral/transdermal dopaminergic substitution therapy, with seven such studies identified [19–25]. One of these studies [21] investigated both STN and GPi targets without separating them in the data analysis; hence, this study is not considered for further evaluation. Similarly, another study [22] mixed GPi and STN, with 4 out of 178 patients receiving GPi-DBS. For GPi as a target, there are only comparative studies with STN [26, 27]. No studies were identified that tested VIM as a target against medication therapy in PD.

***Results:***

***General outcome:*** Comparative studies between STN-DBS and oral/transdermal replacement therapy showed significant improvements in ADL and QoL in favor of STN-DBS. These findings were accompanied by improvements in MFs well as an increased daily On time and decreased daily Off time while daily levodopa dosage was markedly reduced [19].

***Specific predictors of treatment success***: Generally, DBS effectively alleviates levodopa-responsive symptoms of PD, whereas symptoms unresponsive to levodopa are typically not improved by DBS, except for PD tremor, which often responds better to DBS than medication alone [19]. Levodopa-unresponsive symptoms in PD include motor and non-motor issues that improve less well with dopaminergic therapies, presumably due to non-dopaminergic pathways or advanced neurodegeneration. Motor symptoms include postural instability, gait disorders, speech and swallowing difficulties, and dystonia. Certain symptoms, such as freezing of gait (FOG), can exhibit different responses to levodopa treatment. For example, FOG may be levodopa-responsive (“Off freezing”) or levodopa-unresponsive (“On freezing”). Distinguishing between these subtypes is essential for evaluating their potential improvement with DBS. Non-motor symptoms encompass cognitive decline, autonomic dysfunction (e.g., orthostatic hypotension, constipation), sleep disturbances, sensory issues like pain, and neuropsychiatric symptoms such as apathy and depression. Since levodopa responsiveness is a strong predictor of symptom improvement with DBS, the inclusion criteria for STN-DBS include insufficiently controlled MFs and significant improvement in motor symptoms, demonstrated by a standardized levodopa challenge with at least a 33% improvement on the UPDRS-III [19]. Based on these results, further studies explored whether younger patients (i.e., < 60 years) with at least 4 years disease duration, MFs of less than 3 years and at least 50% motor improvement in a standardized levodopa challenge may also benefit from STN-DBS [20, 24]. These patients showed similar effect sizes as compared to the former studies [28]. Poorer preoperative scores on QoL scales in PD patients with shorter disease duration predicted better postoperative outcomes [28], as did a better preoperative response to levodopa [29]. Cognitive and apathy scales remained unaffected, with a potential positive impact on depression [29]. Patients > 70 years of age were typically excluded from the studies. Although the absolute age did not definitively impact postoperative outcomes, it is to suggest that the biological age should guide therapy decisions [28]. Exclusion criteria such as dementia (Mattis score > 130), uncontrolled psychosis/hallucinations or depression, suicidal ideations, and neurosurgical contraindications must be carefully considered [30, 31]. High incidence of suicidal thoughts or acts were reported, but rates did not differ between treatment groups, suggesting these may not be treatment-related effects. Procedural adverse events were nominally higher in the DBS group (~ 25%) compared to standard therapy groups [30, 31].

**Table:** 

Based on the available evidence, the following recommendations were agreed upon in the German PD Guideline: STN-DBS should be offered to patients with PD experiencing MFs with and without dyskinesia that cannot be adequately treated with conservative medication, provided there is at least a 33% improvement in motor symptoms using a standardized levodopa challenge STN-DBS should also be offered to PD patients younger than 60 years and MFs of less than 3 years with at least 4 years disease duration and at least 50% motor improvement in a standardized levodopa challenge DBS is associated with a surgical procedure, and therefore entails special risks, which must be individually weighed against the benefits of the therapy	
**Consensus strength:**	**96.2%, strong consensus**

***Sustainability of clinical symptom control in PD management with MFs, with and without dyskinesias with DBS (STN, GPi, VIM) compared to oral/transdermal standard therapy in PD treatment***

***Rationale:*** Initially, DBS often results in a profound improvement of PD symptoms. This section explores the sustainability of symptom control with DBS (STN, GPi, VIM) compared to oral and transdermal therapies, evaluating sustained outcomes in patients with MFs.

***Evidence base:*** The available randomized controlled studies on the effectiveness of STN-DBS compared to conservative medical therapy cover observation periods ranging from 3 to 24 months [19–25]. Several long-term open-label follow-up studies are summarized in various meta-analyses of STN-DBS [32, 33]. In addition, there is a follow-up study of 51 patients [63]. These open-label follow-ups mainly compare the effects of STN-DBS to the preoperative status in medication resp. motor Off rather than to a solely medically treated control group observed during the study period. For GPi-DBS, two long-term open-label follow-up studies have been identified, documenting outcomes over 5–6 years postoperatively [34, 35]. Regarding the randomized controlled comparison studies of STN-DBS versus GPi-DBS [26, 27], open-label follow-ups extend to 3 years each [36, 37]. Regarding VIM-DBS, a long-term uncontrolled open-label follow-up study involving 38 patients has been identified, comparing motor functions and activities of daily living (UPDRS II and Schwab and England Scale) preoperatively to 6 years postoperatively [38].

***Results:*** The mentioned meta-analyses encompassed 8 studies over 5 years involving 273 patients and 3 studies spanning 8–10 years with 52 patients. Throughout these studies, there was a consistent improvement in UPDRS II (ADL) and III (motor function including tremor), although parameters like rigidity, bradykinesia, gait, and dopaminergic medication worsened over time compared to preoperative baseline conditions. Despite the decline in certain motor symptoms, dyskinesias were minimally progressive, and rest tremor remained adequately controlled even after 8–10 years of follow-up. However, axial symptoms deteriorated compared to the preoperative medication Off state, with speech functions showing decline as early as 5 years post-surgery. Another recent meta-analysis [32] expanded on these findings, adding 7 additional studies involving a total of 551 patients over 5 years, with 93 patients followed for 8–11 years. Out of an initial 923 patients, 372 were unavailable for follow-up due to various reasons, including mortality unrelated to DBS. Despite these challenges, the meta-analysis concluded that STN-DBS can sustain motor function improvements for at least 10 years, with notable improvements in dyskinesias and MFs. Moreover, predictors of favorable long-term outcomes with STN-DBS were identified, including accurate electrode placement within the sensorimotor region of the STN, younger age (< 65 years) at the time of surgery, higher baseline UPDRS scores in the Off state, severe MFs and significant gait disturbances during the Off medication period. Conversely, older age (> 65 years), particularly impacting axial motor functions, and longer disease duration were associated with poorer long-term prognosis in terms of ADL. Regarding GPi-DBS, the available long-term data remain limited due to small sample sizes. Nonetheless, initial findings suggest sustained reductions in UPDRS III scores (motor function) post-surgery compared to preoperative Off medication states, with continued improvements noted in dyskinesia control even after 5 years. However, several patients initially receiving GPi-DBS later underwent STN-DBS due to inadequate symptom control, highlighting variability in response.

***Significance of GBA mutations***: Evidence regarding the effects of DBS in patients with genetic mutations, such as Glucocerebrosidase A (*GBA*) is evolving, with studies highlighting both benefits and challenges. Pal et al. analyzed cognitive trajectories in GBA mutation carriers with and without STN-DBS [39]. Their findings suggest that the combination of GBA mutations and STN-DBS may accelerate cognitive decline. GBA^+^DBS^+^ patients exhibited greater cognitive decline compared to both non-GBA carriers and GBA carriers without DBS, emphasizing the interaction between genetic predisposition and DBS on cognition. Avenali et al. examined long-term outcomes in a large Italian cohort, showing that GBA mutation carriers (GBA-PD) experience significant motor improvement and reduced motor fluctuations, dyskinesias, and impulsive-compulsive disorders post-DBS. However, cognitive decline became apparent after 3 years, with dementia rates at 5 years higher in GBA-PD (25%) than non-GBA-PD (11%) [40]. These studies highlight the potential of DBS as an effective treatment for motor symptoms of GBA-PD, but also emphasize the need for careful cognitive monitoring. They open the discussion for pre-surgical genetic screening to provide patients with more individualized counseling regarding their expected clinical outcome with DBS therapy.

**Table:** 

Based on the available evidence, the following recommendations were agreed upon in the German PD Guideline: Current data indicate that STN-DBS is effective in reducing dyskinesia, MFs, rigidity, and tremor, as well as in decreasing dopaminergic substitution for at least 10 years. However, there is no evidence of delaying symptoms attributed to progressive neurodegeneration (e.g., dementia, axial symptoms, segmental akinesia) Comparable long-term data are not available for GPi-DBS VIM-DBS should not be used in the treatment of PD with MFs and dyskinesia	
**Consensus strength:**	**100%, strong consensus**

***Comparative effectiveness and safety of DBS (STN, GPi, VIM) vs. oral/transdermal standard therapy in treating PD with pharmacoresistant tremor***

***Rationale:*** Tremor in PD often shows a limited response to oral medications. This section assesses the effectiveness and safety of DBS (STN, GPi, VIM) compared to oral and transdermal therapies.

***Background:*** PD tremor includes any pathological tremor in PD patients, with resting tremor occurring in about 75% of patients and many also experiencing action tremors.

***Evidence base:*** Randomized, controlled studies comparing DBS with best medical treatment (BMT) are only available for STN-DBS. Six large RCTs and one smaller pilot RCT have been identified [19–25]. Only two RCTs [20, 22] provide data specifically on the efficacy concerning PD tremor. Studies on the efficacy of GPi-DBS compared to BMT for pharmacoresistant PD tremor do not exist. The efficacy of VIM-DBS for pharmacoresistant PD tremor has only been investigated in uncontrolled studies.

***Results:*** Specific efficacy data on PD tremor are detailed in only two RCTs [20, 22]. The PD-SURG study [22] found a significant improvement in PDQ-39 scores with STN-DBS plus BMT compared to BMT alone, particularly among patients primarily treated for tremor. In a pilot RCT [20], STN-DBS demonstrated marked tremor improvement over 6 to 18 months compared to BMT. VIM-DBS for pharmacoresistant PD tremor has been explored primarily through uncontrolled studies [41–45], indicating substantial tremor reduction and sustained benefits up to 21 years postoperatively.

**Table:** 

Based on the available evidence, the following recommendations were agreed upon in the German PD Guideline: STN-DBS should be offered to patients with PD who have severe tremor that cannot be adequately treated with conservative medication. Bilateral placement is preferable Unilateral or bilateral VIM-DBS and GPi-DBS are effective for PD tremor that cannot be controlled with medication and should be considered when STN-DBS is contraindicated The therapy involves a surgical procedure, and therefore carries specific risks that must be carefully balanced against its potential benefits	
**Consensus strength:**	**92.3%, consensus**

***Sustainable clinical symptom control of DBS (STN, GPi, VIM) vs. oral/transdermal standard therapy in PD with pharmacoresistant tremor***

***Rationale*:** Achieving long-term control of pharmacoresistant tremor in PD is a critical challenge. This section examines the durability of clinical benefits with DBS (STN, GPi, VIM) compared to oral and transdermal therapies, providing insights into sustained management outcomes.

**Background:** DBS in the STN, GPi, and VIM target areas is effective for treating medication-resistant tremors in PD; this section aims to clarify how sustainable this clinical symptom control is compared to medication therapy.

**Evidence base:** There are no randomized controlled long-term data available on this issue. Regarding the sustainability of DBS in the STN, GPi, and VIM targets, only uncontrolled follow-up observations exist, which do not compare against standard medication therapy but rather against preoperative symptom severity in the medication Off state (STN: [35, 46, 47]; GPi: [34, 35]), as well as comparing GPi-DBS versus STN-DBS [36, 37], and for VIM comparing DBS On versus DBS Off, or against preoperative clinical status [38, 48–50].

**Results:**

***STN:*** In two summaries of identical studies on open long-term data evaluating the efficacy of STN-DBS [46, 47], 8 studies with a 5-year follow-up (273 patients) and 3 studies with an 8–10-year follow-up (52 patients) are reported. Tremor control remained stable over the observation period, with an average reduction of approximately 80% compared to the preoperative state. In another recent meta-analysis [32], 5 additional studies were identified, totaling 477 patients with a 5-year follow-up, where tremor severity was separately assessed. Of these, 93 patients were clinically followed for 8–11 years, showing an average reduction in tremor severity of 73% after 5 years and 74% after 8–11 years, relative to the preoperative medication Off state. ***GPi:*** For the efficacy of GPi-DBS, long-term data are limited with very small sample sizes: 6 patients (initial cohort 11 patients) [34] and 16 patients [35]. One study did not show significant tremor reduction after 3 years (nine patients) or 5 years (six patients) compared to DBS Off assessment points. In another study, tremor in the medication Off/DBS On state remained significantly reduced after 5–6 years (65.5% improvement) compared to the preoperative medication Off state. Comparative studies between STN-DBS and GPi-DBS [36, 37] only separated out tremor control in one study [36]. It showed no difference between STN and GPi in tremor control after 6 months in the medication Off/DBS On state, which remained stable over 36 months (blinded for STN vs. GPi, not for DBS Off vs. On).

***VIM:*** For VIM-DBS, four studies were identified with open follow-ups ranging from 12 months to 21 years [38, 48–50]. Significant tremor suppression effects were consistently described: unilateral—67% improvement after 1 year, 85% after 5 years, 58% after 11–15 years, and 63% after 16–21 years; bilateral—73% after 1 year, 64% after 6–7 years, 69% after 11–15 years, and 60% after 16–21 years. Short-term improvements in daily activities were noted for only about 1-year postoperatively. Stimulation-related side effects were attributed to electrode positioning in the target area (up to 45% paresthesia, up to 41% pain, up to 75% dysarthria, and up to 93% gait and balance disturbances), often reversible with DBS parameter adjustments. Balanced stimulation programming in the tremor-sensitive regions of VIM and the posterior subthalamic area (cZI; PSA) is typically required for achieving optimal tremor suppression without directly related adverse effects (ataxia, dysarthria, paresthesia).

**Table:** 

Based on the available evidence, the following recommendations were agreed upon in the German PD Guideline: STN-DBS remains effective in the long term, with relevance for at least 10 years in treating pharmacoresistant PD tremor. It should be offered to patients with pharmacoresistant PD tremor, considering contraindications, and preferably performed bilaterally GPi-DBS provides sustainable symptom control for PD tremor. The choice of target area in patients with PD and medically uncontrollable tremor should consider their individual symptom profile Uni- or bilateral VIM-DBS is effective in the long term for medically uncontrollable Parkinson’s tremor and may be considered in cases where STN-DBS or GPi-DBS are contraindicated	
**Consensus strength:**	**100%, strong consensus**

***Comparative effectiveness of DBS targets (STN, GPi, VIM) in treating PD with MFs, with and without dyskinesias***

***Rationale:*** This section compares the effectiveness of DBS targeting the STN, GPi, and VIM in managing MFs in PD in patients with and without dyskinesias to determine the most effective approach for symptom control.

***Background:*** Electrodes can be implanted in the STN, GPi, or VIM. The choice of target is individualized based on the primary symptoms and any specific contraindications.

***Evidence base:*** Randomized and controlled studies allowing for a comparison of effectiveness between these targets are only identified for STN-DBS versus GPi-DBS [26, 27].

***Results:*** In two RCTs [26, 27], patients were evenly assigned between target points: 299 and 125 patients were followed up for 2 years and 1 year, respectively. The NSTAPS study [27], conducted in the Netherlands, focused on primary endpoints including ADL and a composite score for cognition, mood, and behavior, showing no significant differences between groups after 12 months. However, secondary outcomes suggested potential superiority of STN-DBS over GPi-DBS in reducing motor symptoms and improving ADL during medication Off states. GPi-DBS demonstrated better control of dyskinesias during medication On states, with no disparities noted in secondary measures like daily on-time without disabling dyskinesias. In the CSP486 study [26], the primary endpoint was the change in motor function (UPDRS III) due to DBS without medication after 24 months, which did not differ between STN-DBS and GPi-DBS groups. Secondary outcomes such as self-assessment of daily functions, QoL, cognitive functions, and side effects also showed no differences. However, the improvement in motor symptoms with STN-DBS was modest at around 26%, contrasting with approximately 50% improvements seen in other studies. Possible reasons include lower preoperative motor symptom improvement with dopaminergic medication or target blinding during postoperative care. STN-DBS achieved higher stimulation effectiveness along with reduced dopaminergic medication, reflected in a 43% reduction (NSTAPS study) or 31% reduction (CSP486 study), respectively. Complication rates from surgery or therapy did not differ significantly between targets. VIM-DBS for PD with MFs and dyskinesias lacks evidence from controlled studies, relying only on findings from uncontrolled investigations [41–45], where improvements in symptoms like akinesia, rigidity, and dyskinesias have not been demonstrated.

**Table:** 

Based on the available evidence, the following recommendations were agreed upon in the German PD Guideline: STN-DBS should be preferred over GPi-DBS in the differential therapeutic considerations for PD with MFs with and without dyskinesias VIM-DBS should not be used in the treatment of PD with MFs with and without dyskinesias DBS involves a surgical procedure and associated risks that must be weighed individually against the potential benefits of the therapy	
**Consensus strength:**	**100%, strong consensus**

***Comparative effectiveness of DBS (STN, GPi, VIM) in treating PD with pharmacoresistant tremor***

***Rationale:*** Selecting the appropriate surgical target is crucial for optimizing therapy outcomes. This section examines the effectiveness of DBS targeting the STN, GPi, and VIM in treating pharmacoresistant tremor in PD, comparing outcomes across these different DBS targets.

**Evidence base:** A systematic review with meta-analysis [51] compared the efficacy of STN-DBS and GPi-DBS in the treatment of PD tremor, incorporating five RCTs [27, 36, 52–54]. Randomized controlled studies comparing the efficacy of STN-DBS versus VIM-DBS do not exist.

**Results:** In a meta-analysis [51], the efficacy of STN-DBS (*N* = 263) and GPi-DBS (*N* = 226) for Parkinson’s tremor was analyzed over a period of up to 60 months across 489 patients from 5 randomized studies [27, 36, 52–54]. Additional unpublished data were obtained from two RCTs [27, 52] via the principal investigators and included in the analysis. The five RCTs underwent a random-effects model meta-analysis. A moderator variable analysis was conducted to assess differences in treatment effects between STN-DBS and GPi-DBS. In the overall comparison of DBS On versus DBS Off, a significant standardized mean difference of 0.36 indicated that DBS reduces PD tremor with a moderate effect size. The moderator variable analysis comparing STN-DBS versus GPi-DBS revealed two significant standardized effect sizes: 0.38 for STN-DBS and 0.35 for GPi-DBS, which were not significantly different. However, across all five studies, STN-DBS tended to show a slightly stronger tremor-suppressing effect compared to GPi-DBS. Thus, the results of the meta-analysis indicate that there are no statistically significant differences between STN-DBS and GPi-DBS in the long-term improvement of Parkinson’s tremor. Randomized controlled studies comparing the efficacy of STN-DBS versus VIM-DBS do not exist. The efficacy of VIM-DBS for pharmacoresistant tremor has only been examined in uncontrolled studies (see above).

**Table:** 

Based on the available evidence, the following recommendations were agreed upon in the German PD Guideline: STN-DBS and GPi-DBS are equally effective in treating medication-resistant PD tremor. Therefore, the choice of target area for patients with PD and medication-resistant tremor should be made based on their overall individual symptom profile Unilateral or bilateral VIM-DBS is effective for medication-resistant PD tremor in both, short- and long-term, and can be considered if there are contraindications for STN-DBS or GPi-DBS	
**Consensus strength:**	**96.2%, strong consensus**

***Effectiveness of DBS (STN, GPi) in treating non-motor symptoms and their fluctuations (sleep, pain, autonomic symptoms (excessive sweating, dysuria, gastrointestinal symptoms, orthostatic hypotension), neuropsychiatric symptoms (apathy, depression, anxiety, impulse control disorders, punding, dopamine dysregulation syndrome) compared to oral/transdermal standard therapy in PD***

***Rationale:*** This section examines the effectiveness of DBS (STN, GPi) in managing non-motor symptoms and their fluctuations, including sleep disturbances, pain, autonomic dysfunction, and neuropsychiatric symptoms, compared to oral and transdermal therapies in PD.

***Background:*** Neuropsychiatric fluctuations, which affect autonomic and sensory functions alongside motor and cognitive abilities, are common in PD but often overlooked due to limited assessment tools. Nevertheless, DBS, particularly STN-DBS, is widely used for motor complications and has shown some effects on mood, pain perception, and autonomic functions.

***Evidence base and results***

***Depression***: RCTs comparing DBS with medication suggest DBS does not significantly alter depression outcomes compared to medication alone [19–25]. Initial reports indicated higher suicide risks among DBS patients, but subsequent studies found no significant differences in suicidal tendencies compared to the general PD population [30, 31].

***Apathy*:** Meta-analyses indicate a potential worsening of apathy under DBS compared to pre-surgical and medical therapy conditions [55]. Drapier et al*.* reported worsening of apathy under STN-DBS compared to a control group [56], while Valldeoriola et al*.* found no difference in apathy between STN-DBS and patient receiving levodopa/carbidopa intestinal gel (LCIG) [57].

***Neuropsychiatric fluctuations*:** DBS shows promise in reducing neuropsychiatric fluctuations, benefiting patients with early motor complications [58, 59].

***Impulse control disorders (ICD)*:** DBS may alleviate hyperdopaminergic behaviors and some forms of impulse control disorders [58–60], possibly because of reducing dopaminergic drug dosing. However, studies focusing solely on severe ICD cases are lacking [61].

*Sleep*: DBS improves overall sleep quality and reduces daytime fatigue compared to baseline and medical therapy controls [62–66].

***Autonomic symptoms*:** DBS shows potential in improving certain autonomic symptoms like dysuria and thermoregulation [62, 67, 68]. Clinical significance and differentiation from primary urological causes remain uncertain.

Pain: Studies suggest DBS provide moderate relief from PD-associated pain, especially Off dystonia [69–71].

**Table:** 

Based on the available evidence, the following recommendations were agreed upon in the German PD Guideline: Non-motor symptoms are currently not established indications for STN-DBS or GPi-DBS However, the presence of NMFs, impulse control disorders, and/or sleep disorders may support the consideration of STN-DBS in PD	
**Consensus strength:**	**100%, strong consensus**

#### Continuous subcutaneous or intrajejunal delivery of dopaminergic drugs via pump therapies

#### Apomorphine

***Effectiveness and safety of subcutaneous apomorphine pump therapy compared to oral/transdermal standard therapy in treating MFs and dyskinesias in PD***

***Rationale:*** This section assesses the effectiveness and safety of subcutaneous apomorphine pump therapy.

***Background:*** Apomorphine is a mixed D1 and D2 agonist, most potent among all dopamine agonists, with only 4% oral bioavailability necessitating subcutaneous administration for full effect. Without requiring active transport mechanisms to reach the CNS, its motor effect, compared to levodopa, begins significantly faster (within 4–12 min) and lasts 45–60 min on average. Continuous subcutaneous infusion via a pump worn externally is used to smooth MFs, typically delivering apomorphine over 12–16 (occasionally up to 24) h into the abdominal or thigh subcutaneous tissue.

***Evidence base:*** Although apomorphine has been used for over 30 years, this literature review identified only one randomized controlled trial and eight longitudinal cohort studies, with one exception having *N* < 50.

***Results:***

***Effectiveness:*** The only randomized controlled trial, the TOLEDO study, examined the efficacy and safety of continuous apomorphine infusion compared to placebo in 106 patients over 12 weeks [69]. The primary endpoint was the absolute reduction in daily Off time. Concomitant medication was reduced if dopaminergic adverse effects (e.g., dyskinesias) occurred. Up to 300 mg oral levodopa was allowed if needed. Apomorphine infusion (mean 4.68 [± 1.50] mg/h) reduced Off time by 37% compared to baseline and significantly by 1.89 [3.2–0.6] h/day compared to placebo (28% more than placebo). Among the secondary endpoints, apomorphine was significantly superior to placebo in the following categories: number of patients with > 2 h reduction in off time/day: − 33.4%; patient global impression of change: − 1.20; on-time without troublesome dyskinesias: − 1.97 h/day; reduction in levodopa equivalent daily dose: − 328.5 mg. Apomorphine was not superior in reducing oral levodopa dose/day, UPDRS III in on state, and QoL. In other uncontrolled, partially multicenter and partly prospective observational studies lasting up to 2 years, reductions in daily Off time ranging from 40 to 80% compared to baseline were observed among patients who continued therapy. Dyskinesias were reported in only some of the studies. Many patients reported overall improvement, and there were indications of a relationship between reduction in oral medication and the extent of dyskinesia improvement [70–75].

***Safety:*** In terms of drug safety, the TOLEDO study found significantly more adverse events in the apomorphine group compared to placebo. The most common adverse events included subcutaneous nodules (44%), nausea and somnolence (each 22%), erythema at the infusion site (17%), dyskinesias (15%), headache (13%), and insomnia (11%). Adverse events led to study discontinuation within the 12-week observation period in 11% and to dose adjustment in 48% of patients [69]. During the open-label 52-week phase, the incidence of subcutaneous nodules increased (54%), while the frequency of other adverse events remained unchanged. The discontinuation rate due to adverse events was 16.7% [71]. In former uncontrolled cohort studies, adverse events were only inconsistently described. The most common were subcutaneous nodules (8–100%), nausea (7–27%), psychosis (8–40%), hypomania/impulse control disorders (3–9%), somnolence (5–67%), symptomatic orthostatic hypotension (16–25%), and hemolytic anemia (1.2–1.5%). In the open-label extension study of the TOLEDO trial over an additional 52 weeks, which included 84 out of the original 106 patients and was completed by 59 patients, Off time was reduced by 53% compared to baseline (− 3.66 h/day) [71].

**Table:** 

Based on the available evidence, the following recommendations were agreed upon in the German PD Guideline: Apomorphine pump therapy should be used for the treatment of MFs to reduce Off phases and dyskinesia and prolong On time Due to the complex handling of this procedure and the frequency of complications, close patient monitoring is recommended, and it should only be started and supervised by physicians experienced in this therapeutic procedure	
**Consensus strength:**	**100%, strong consensus**

***Effectiveness and safety of subcutaneous apomorphine pump therapy compared to oral/transdermal standard therapy in treating non-motor PD symptoms and their fluctuations (sleep, pain, autonomic symptoms (excessive sweating, dysuria, gastrointestinal symptoms, orthostatic hypotension), neuropsychiatric symptoms (apathy, fatigue, depression, anxiety, impulse control disorders, punding, dopamine dysregulation syndrome)***

***Rationale:*** This section evaluates the effectiveness and safety of subcutaneous apomorphine pump therapy in treating non-motor symptoms such as sleep disturbances, pain, autonomic, and neuropsychiatric symptoms, highlighting its role in advanced PD care.

***Background:*** Apomorphine, despite being the strongest and fastest-acting dopamine agonist with a short half-life, may exacerbate complications, particularly in patients with orthostatic hypotension, impulse control disorders, or a history of psychosis. However, it can also be beneficial, especially in managing affective disorders associated with advanced PD.

***Evidence base:*** There are no randomized controlled trials or large cohort studies addressing this question. Three longitudinal observational studies and one review examined the effects of subcutaneous apomorphine infusion on the Non-Motor Symptom Scale (NMSS) compared to LCIG or DBS.

***Results:*** NMSS data from the TOLEDO study [69] have not yet been published. Several open observational and case-based studies involving a total of 93 patients have shown that subcutaneous apomorphine infusion can have favorable effects on both the NMSS total score and specific non-motor subdomains [64, 72, 73]. In summary, over a treatment period of 6–12 months, the NMSS total score improved across all domains, with particular emphasis on the domains of sleep/fatigue, mood/apathy, attention/cognition, perception/hallucinations, attention/memory, and other symptoms. The least effects were observed in the cardiovascular and sexual function domains. Notably, LCIG showed relatively larger effects in almost all domains in the EuroInf studies [64, 72, 73]. Although apomorphine, as a potent D1 and D2 receptor agonist, inherently carries a greater risk of hallucinations compared to levodopa, favorable effects in reducing mild visual hallucinations have been reported with apomorphine infusion. Possible reasons for this include reduction in concomitant medication, including polypharmacy, younger patient age, and shorter disease duration (62.2 years and 13.5 years, respectively). In addition, a potential favorable psychotropic effect of apomorphine, based on its structural similarity to piperidine, has been postulated [76].

**Table:** 

Based on the available evidence, the following recommendations were agreed upon in the German PD Guideline: Continuous subcutaneous apomorphine infusion can alleviate non-motor symptoms measured in the NMSS (sleep/fatigue and mood/apathy, attention/cognition as well as perception/hallucinations and attention/memory and other symptoms) These effects can be used as possible determinants in the selection of patients for apomorphine infusion therapy	
**Consensus strength:**	**95.3%, strong consensus**

***Sustainability of clinical symptom control: subcutaneous apomorphine pump therapy vs. standard oral/transdermal therapy in PD with MFs, including dyskinesia***

***Rationale:*** This section examines the long-term sustainability of symptom control with subcutaneous apomorphine pump therapy compared to standard oral and transdermal therapies in managing MFs, including dyskinesias, in advanced PD.

***Background:*** The transition to an invasive therapy represents a significant change from previous oral medication for the patient, aiming for sustainability, with subsequent evaluation focusing on its long-term effectiveness and safety.

***Evidence base:*** One randomized controlled study with an observation period of 18 months was found, along with eigth longitudinal cohort studies.

***Results:*** In the open-label phase of the TOLEDO study, which included an observation period of 18 months, the mean reduction in Off time was − 3.66 h (− 45%), with moderate reduction in bothersome dyskinesias. The oral levodopa dose was reduced by approximately 25%, and levodopa equivalent dose by about one third. 30% of participants did not complete the study, with 17% discontinuing due to adverse events such as skin reactions, fatigue, autoimmune hemolysis, delirium, dementia, attention deficit, lymphoma, nausea, panic attacks, and somnolence [77]. Larger long-term studies are lacking. Prospective open-label studies mostly involved small patient numbers (< 50 patients) with observation periods ranging from at least 1.5 years (average 40 months) [64, 71, 73, 76, 78, 79]. They describe a stable motor effect with Off time reduction of 25–50% compared to baseline, but dropout rates ranged from 20 to 83%. An open-label study involving 114 patients treated with subcutaneous apomorphine infusions for at least 6 months examined reasons for treatment discontinuation [80]. The mean duration until discontinuation was 2.42 ± 2.23 years (0.5–9.2). Severe dyskinesias recurring in 38% were the primary reason for discontinuation; about 16% (mostly elderly patients) stopped due to cognitive difficulties, approximately 14% due to skin reactions, about 12% due to postural instability, and 11% each due to hallucinations or depression/anxiety. The longest prospective study over a 5-year period included 12 patients (average age at study start 58 years, disease duration 9 years): Only 2 of 12 patients (17%) were still receiving apomorphine after 5 years. The mean treatment duration was 30 months. Three patients had died between years 2–5 (not related to treatment), 5 discontinued treatments between years 3–5 due to recurrence of severe fluctuations (severe dyskinesias and Off periods) and subsequently received DBS or LCIG, 2 discontinued in the second year due to skin nodules, and 1 was lost to follow-up. Among those who continued treatment, Off periods remained controlled, but the duration and disability due to dyskinesias did not significantly improve [81].

**Table:** 

Based on the available evidence, the following recommendations were agreed upon in the German PD Guideline: The efficacy of subcutaneous apomorphine pump therapy for the treatment of MFs has been proven for a period of 18 months, in individual cases up to 5 years The risk of treatment discontinuation increases with increasing treatment duration (on average after 2.5 years)	
**Consensus strength:**	**95%, consensus**

#### Levodopa–carbidopa intestinal gel

***Effectiveness and safety of levodopa–carbidopa intestinal gel pump therapy compared to oral/transdermal standard therapy in treating MFs and dyskinesias in PD***

***Rationale:*** This chapter examines the clinical utility, efficacy, and safety profile of levodopa–carbidopa intestinal gel (LCIG) therapy for PD, highlighting its ability to provide continuous dopaminergic stimulation and reduce motor fluctuations, while addressing the potential complications and adverse events associated with its use.

***Background:*** LCIG is a highly concentrated formulation of levodopa and the decarboxylase inhibitor carbidopa (20/5 mg/ml) infused directly into the jejunum via a pump system, achieving steady plasma levels for continuous stimulation of striatal dopamine receptors. The system includes a portable pump, a cassette containing 100 ml of LCIG (equivalent to 2000/500 mg levodopa/carbidopa), and a percutaneous endoscopic gastrostomy with jejunal extension (PEG-J). In some preparations, the catechol-O-methyltransferase inhibitor entacapone is included, and this preparation is referred to as LECIG. Treatment initiation typically necessitates a hospital stay, often starting with a naso-jejunal tube phase to assess efficacy before proceeding to PEG-J placement.

***Evidence base:*** In total, 2 randomized controlled trials and 18 longitudinal larger cohort studies were identified.

***Results:***

***Effectiveness:*** Two randomized controlled trials examining the motor effect of LCIG were identified [82, 83]. A Swedish multicenter randomized controlled crossover study involving 21 patients over 3 weeks showed a significant reduction in severe Off periods with LCIG compared to oral therapy. There was no difference in the occurrence of dyskinesias. UPDRS part II and IV were significantly better in the LCIG group, while part III during motor On was unchanged [82, 83]. The highest quality study was a double-blind, double-dummy randomized controlled trial over 12 weeks with 37 patients in the LCIG group and 34 patients on oral therapy. Off periods in the LCIG group were reduced from 6.3 to 2.3 h/day (64% reduction) and significantly better by −1.91 h/day compared to oral therapy. On time without troublesome dyskinesias was significantly greater than in the oral group. UPDRS part II was significantly reduced in the LCIG group compared to orally treated patients, while UPDRS part III was unchanged in motor On [84]. Several open-label studies confirm efficacy in reducing On time without troublesome dyskinesias [73, 85–89, 97]. Improvement and stability are maintained in several studies over at least 12 months. Most open-label studies lacked a control group, and many were retrospective or did not recruit patients consecutively.

***Safety****:* Regarding drug safety, in the placebo-controlled double-dummy study, adverse events occurred in 95% of the LCIG group and 100% of the comparator group; the majority occurred within the first weeks, with nearly all (89%) associated with PEG-J insertion (abdominal pain, nausea, constipation, flatulence, erythema at the incision site). Three patients discontinued the study within the 12-week observation period due to adverse effects: one in the LCIG group (psychosis) and two in the comparator group (peritonitis/pneumonia and wound secretion) [84]. The open-label GLORIA registry, following 208 patients on a stable treatment regimen for 24 months, documented that 69% experienced at least one adverse event, 45% at least one serious event, and 10% discontinued treatment due to a serious event. Serious events leading to treatment discontinuation included PEG-J-associated problems (3%), neuropsychiatric complications (1%), and polyneuropathies (0.5%). Other adverse events were disease-related (aspiration, pneumonia, disease progression) or attributable to other conditions (intracranial bleeding, bile duct carcinoma). PEG-J complications such as dislocations, occlusions, pump malfunctions, stoma infections occurred in 21%, neuropsychiatric complications (delirium, hallucinations, depression) in 10%, weight loss in 6%, and polyneuropathies in 11.5% [98]. The cause of newly occurring polyneuropathies under LCIG, with an incidence exceeding 10% in other series, is not conclusively clarified. Several studies have observed a decline in vitamin B6, B12, and folate levels during LCIG, which are substrates in levodopa metabolism [99–102]. Particularly, vitamin B6 rapidly declines with high doses of LCIG [102].

**Table:** 

Based on the available evidence, the following recommendations were agreed upon in the German PD Guideline: LCIG can significantly increase On time without troublesome dyskinesias and significantly reduce Off time; thus; it should be used to treat MFs inadequately controlled orally LCIG treatment is relatively safe, with the most common complications being associated to PEG-J Prior to treatment initiation, electrophysiological neuropathy screening and assessment of vitamins B6, B12, and folate levels, as well as body weight, should be conducted and monitored during treatment, and substituted if necessary Due to the complexity of this procedure and the frequency of complications, close patient monitoring is recommended, and initiation and management should only be undertaken by physicians experienced in this therapy	
**Consensus strength:**	**100%, strong consensus**

***Effectiveness of levodopa–carbidopa intestinal gel pump therapy compared to oral/transdermal standard therapy in treating non-motor symptoms and their fluctuations in PD***

***Rationale:*** This section assesses the effectiveness of LCIG pump therapy compared to oral and transdermal therapies in managing non-motor symptoms and their fluctuations in advanced PD.

***Background:*** The frequency and severity of non-motor symptoms increase throughout the course of PD, significantly impacting QoL. LCIG therapy may affect these non-motor symptoms associated with PD.

***Evidence base:*** In total, 12 longitudinal larger cohort studies and 2 review articles were identified.

***Results:*** Until 2015, eight open-label studies confirmed that LCIG reduces the total score of NMSS over treatment periods ranging from 6 to 25 months, with specific positive effects on sleep and autonomic dysfunction, particularly gastrointestinal symptoms [73, 87, 90, 93, 96, 97, 103, 104]. Recent review articles have also confirmed LCIG’s generally positive effect on non-motor symptoms [105, 106]. Studies included in these reviews were the GLORIA registry which demonstrated beneficial effects of LCIG on sleep disturbance, apathy, and gastrointestinal dysfunction in NMSS after 24 months of treatment [85], and the interim analysis of the DUOGLOBE study showing overall improvement in NMSS total score after 6 months [18]. Other open-label studies with a 6-month observation period found improvements in the overall NMSS score, with specific effects in domains such as cardiovascular symptoms, attention/memory, urological symptoms [73, 93]. According to data from the GLORIA registry, the baseline NMSS score can predict the non-motor response to LCIG treatment after 2 years of treatment [107]. LCIG demonstrated greater effects in almost every domain compared to a non-randomized cohort receiving apomorphine infusions in the EuroInf studies [64, 73].

**Table:** 

Based on the available evidence, the following recommendations were agreed upon in the German PD Guideline: LCIG can improve non-motor symptoms such as sleep disturbances, apathy, gastrointestinal dysfunction, cardiovascular symptoms, attention/memory, urological symptoms in PD patients with orally uncontrollable MFs These effects can be considered as potential determinants when selecting patients for LCIG treatment	
**Consensus strength:**	**100%, strong consensus**

***Long-term clinical symptom control of levodopa–carbidopa intestinal gel pump therapy compared to oral/transdermal standard therapy in PD with MFs including dyskinesias***

***Rationale:*** This chapter reviews the long-term efficacy and safety of invasive therapies for PD, and discusses challenges such as treatment discontinuation due to device-related issues, cognitive decline, or lack of efficacy in axial symptoms.

***Background:*** Invasive therapies represent a significant change for patients from their previous oral medication and should ideally be sustainable, so that the studies on long-term efficacy and safety are summarized here.

***Evidence base:*** In total, 15 longitudinal larger cohort studies were identified.

***Results:*** Randomized controlled long-term studies do not exist. Open studies involving cumulatively > 600 patients over at least 12–24 months report a stable effect in reducing Off time and On time with disabling dyskinesias without significant increase in MFs over this period [18, 85, 87, 88, 91, 92, 95, 96, 98, 103, 108]. The most common reasons for discontinuation of treatment are recurrent PEG-J dislocations [109, 110], primarily occurring in delirious or demented patients [111]. Approximately 35–50% of treatment discontinuations occur within the first year. In an Italian study of 905 patients with a mean treatment duration of 6 years, an overall discontinuation rate of 25.7% was found, with 9.5% discontinuing within the first year. Reasons for discontinuation within the first year were predominantly lack of effectiveness on axial symptoms (gait disturbance and falls) [112]; additional factors for early discontinuation included socio-medical factors (living alone) [111]. The most common reasons for late treatment discontinuation were PEG-J-related issues (stoma infections/tube dislocations), difficulties operating the system by elderly patients/family members, and cognitive decline associated with PD [112].

**Table:** 

Based on the available evidence, the following recommendations were agreed upon in the German PD Guideline: LCIG is effective in the long-term reduction of motor fluctuations and should be used as a permanent treatment The average duration of treatment in large long-term trials is 6 years Before starting treatment, patients should be given detailed information about the expected effects of treatment, particularly with regard to non-dopa-responsive symptoms such as postural instability, falls, and ON freezing	
**Consensus strength:**	**94.5%, consensus**

#### Ablative therapies

***Efficacy and safety of ablative procedures (thermal coagulation, Gamma/CyberKnife, focused ultrasound) versus oral/transdermal standard therapy in treating PD motor symptoms***

***Rationale:*** This chapter provides a comprehensive overview of ablative procedures for PD, highlighting their evolution, evidence base, and limitations, and comparing them to DBS, which remains the preferred treatment due to its established efficacy, safety, and adjustability, while emphasizing MRgFUS as a promising but still emerging therapy option for select cases.

***Background:*** Ablative procedures have in common that they induce localized lesions in specific brain structures such as the VIM, STN, or GPi. Historically used extensively for advanced PD, popularity of these procedures declined with the advent of DBS [113]. Radiofrequency-induced heating and stereotactic gamma radiation were initial methods, with MRgFUS now emerging as a less invasive alternative. MRgFUS allows precise targeting and real-time temperature monitoring, minimizing side effects compared to previous methods like radiofrequency and radiation [114–116].

***Evidence base:*** In contrast to DBS, which has been extensively studied with comparative trials [29, 36, 117], randomized trials directly comparing drug therapy with ablative procedures are scarce. Randomized, controlled trials for radio frequency thermolesion pallidotomy exist, involving 36 and 37 patients, respectively [118, 119]. However, there are no randomized studies for thalamotomy or subthalamotomy using radiofrequency ablation. MRgFUS thalamotomy has been investigated in both controlled studies [120] and case series [121, 122], focusing on tremor-dominant PD. Recently, separate randomized, double-blind controlled trials have explored MRgFUS-guided unilateral STN lesion and unilateral pallidotomy in unilateral dominant PD [123, 124]. Direct comparisons between DBS and ablative procedures are limited. For instance, Schuurman et al. demonstrated that both DBS of the VIM and thalamotomy are similarly effective in controlling medication-resistant tremors, with DBS showing fewer side effects and greater functional improvement. However, data regarding pallidotomy and subthalamotomy are insufficient to determine their comparative effectiveness and safety against DBS. There is also no evidence suggesting a preference for ablative procedures for specific patient groups. Due to better controllability and a clearer understanding of its effects and side effects [29, 36, 117], DBS remains the preferred choice over ablative procedures (such as radiofrequency lesion and radiosurgery) for treating PD. A definitive comparison with MRgFUS is currently hindered by insufficient data. A recent meta-analysis suggests that all targeted procedures have comparable tremor-suppressive effects [125]. Controlled, randomized studies exist for MRgFUS ablation of both the STN and GPi [123, 124].

***Results:*** Unlike DBS, which has been extensively compared in studies [29, 36, 117], ablative procedures lack robust randomized comparisons with drug therapies. Pallidotomy, thalamotomy, and MRgFUS thalamotomy have shown efficacy in controlling PD symptoms, especially tremor, with some studies indicating improvements in quality of life and activities of daily living [123, 124]. Long-term data for pallidotomy suggest sustained benefits in treating PD motor symptoms but with higher rates of side effects compared to DBS [126–129]. Studies directly comparing DBS with ablative procedures are sparse. Evidence suggests VIM-DBS and thalamotomy are comparable for tremor suppression, with VIM-DBS associated with fewer side effects and greater functional improvement [130]. However, the overall safety and efficacy of MRgFUS compared to DBS remain unclear due to limited data [29, 36, 117]. DBS offers advantages in adjustability and long-term management compared to ablative procedures, whereas the latter provide irreversible structural changes. While MRgFUS shows promise especially in tremor reduction, particularly for patients unsuitable for DBS, current evidence does not support a clear preference for ablative procedures over DBS [29, 36, 117]. Thus, DBS remains the preferred choice due to its established efficacy and safety profile in PD treatment. MRgFUS in VIM can currently be recommended to a limited extent for unilateral PD with tremor dominance. However, the indication for VIM ablation in PD tremor should be made with caution, as good tremor suppression can also be achieved with DBS or lesion of the STN. Moreover, initially tremor-dominant PD syndromes may change to an equivalent type over the course of the disease which bradykinetic symptoms or MFs will not therapeutically respond to VIM ablation. It is currently unclear whether MRgFUS of the STN improves PD-associated tremor sufficiently and sustainably compared to DBS.

**Table:** 

Based on the available evidence, the following recommendations were agreed upon in the German PD Guideline In contrast to DBS, for which high-quality comparative studies are available, there are few or no randomized studies on ablative procedures for PD. Except in the case of contraindications, DBS should currently be recommended as the first option Thalamotomy and subthalamotomy using radiofrequency ablation are no longer recommended for PD. Treatment using radiosurgical procedures (Gamma-Knife, CyberKnife) is not recommended due to the lack of studies and the potentially high risk of complications Pallidotomy may be considered in advanced PD if there are drug fluctuations that are difficult to control and treatment with DBS or pump therapy is not an option For the treatment of PD tremor with unilateral MRgFUS, there is already approval in Europe for all targets (VIM, STN, GPi) but this procedure should still only be carried out in the context of studies or registries The indication for MRgFUS VIM ablation in PD tremor should be made with caution, as good tremor suppression can also be achieved with DBS or lesion of the STN. Moreover, initially tremor-dominant PD syndromes may change to an equivalent type over the course of the disease which bradykinetic symptoms or MFs will not therapeutically respond to VIM ablation All ablative procedures recommended in this guideline should only be used unilaterally	
**Consensus strength:**	**95.8% to 100%, strong consensus**

### Differential indication

#### Differential indications and contraindications of invasive therapies in the treatment of PD

***Rationale:*** Invasive therapies, such as DBS and pump therapies, offer significant benefits for advanced PD patients who do not respond to oral treatments. However, these therapies are not suitable for all patients. Differential indications and contraindications are crucial for identifying which individuals may relevantly benefit from invasive treatments and who may face increased risks. This section explores the factors that guide the decision-making process, ensuring that invasive therapies are used appropriately based on individual patient characteristics, disease progression, and overall health.

***Background:*** All the procedures described above, particularly DBS and pump therapies, are suitable for managing MFs inadequately controlled with oral medication. Tremor is more effectively controlled with DBS compared to pump therapies. NMS may vary widely in advanced PD with MFs and some of them may even preclude certain invasive procedures. This chapter will present the evidence regarding differential indications, contraindications, and exclusion criteria for the various invasive therapies.

***Evidence base:*** For the evaluation of ***DBS***, there are several controlled, randomized studies against standard (medication-based) treatment [29, 36, 117]. Regarding the assessment of the effectiveness of ***LCIG*** therapy, there are two randomized, controlled studies against standard treatment (best medical treatment, BMT), with one study (DYSCOVER) published only on Clinical Trials [84]. There is an observational study for ***LECIG*** therapy [131]. For CSFLI therapy, there is a controlled, randomized study against BMT [132], showing an improvement in motor fluctuations/dyskinesias comparable to DBS. There are only open-label studies available for direct comparison among pump therapies and between pump therapies and DBS [64, 70, 81, 94, 133–135]. For the comparison of ***LCIG and DBS*** [57, 94, 133, 136], only open-label studies are available: there are non-randomized prospective observational studies comparing ***LCIG with CSAI*** [73] and comparing ***LCIG, LECIG, CSAI, and DBS*** [64]. In addition, there is a randomized, open-label, crossover study comparing ***LCIG and CSAI*** [137]. The evidence for the combination of therapies relies on five mostly retrospective case series that exclusively address combinations of ***DBS with pump therapies***. There are no prospective or controlled studies available.

***Results:*** General or procedure-specific aspects must be considered individually to determine the most effective and well-tolerated procedure. Infusion therapies, for instance, are effective only during administration periods, typically halted at night, whereas DBS and ablative procedures maintain effectiveness throughout day and night. Patient preference is also pivotal. A fundamental consideration in treatment selection is whether dopamine overdose significantly contributes to the patient’s symptom profile (e.g., psychiatric side effects of dopaminergic therapy). Among invasive therapies, only STN-DBS allows for reduction of dopaminergic therapy, potentially favoring DBS in such cases. Conversely, hallucinations, often linked to reduced frontal brain function, can negatively predict long-term DBS outcomes [138]. CSAI and LCIG are equivalent to the effectiveness of DBS in the treatment of motor symptoms, with the exception of tremor with possibly better results from DBS [64, 70, 81, 94, 133–135]. Currently, DBS has the most comprehensive evidence base, including long-term data [139]. The assessment of non-motor symptoms is equally important, as each procedure can affect these symptoms differently [64, 140] (Table 1).

**Table 1:** The influence of different interventions on important cross-dimensional outcome parameters and on motor symptoms

Item	Domain	CSAI	LCIG	LECIG	CSFLI	DBS	MRgFUS (unilateral)
Quality of life (PDQ-39; PDQ-8)	QOL	− [72, 171]	+ [141]	?	?	++ [19, 24]	++ [120, 172, 173]
Activities of daily living (ADL; UPDRS II)	ADL	+ [174]	+ [98]	?	++ [132]	++ [175–179]	++ [123] (STN) n.s [124] (GPi)
Motor function in Off; Med Off; (UPDRS III); Off time	MS	++ [69] + [77]	++ [84]	+ [131]	++ [132]	++ [175–179]	++ [123] (STN) [124] (Gpi) [120] (VIM)
Dyskinesia and fluctuations (UPDRS IV)	MS	++ [69] + [77]	++ [84]	+ [131]	++ [132]	++ [175–179]	++ [124] (Gpi)
Cardiovascular (incl falls/orthostasis)	NMS^3^	+/− [72, 73]	+ [180]	?	?	−	?
Sleep/fatigue	NMS	++ [145] + [64, 72, 73]	+/− [64, 73, 85, 93, 96, 150–152]	?	?	+ [64, 181–183]	?
Mood/cognition	NMS	+ [64, 72, 73]	+/− [64, 73, 93, 96]	?	?	++ [64]	?
Perception problems/hallucinations	NMS	+ [64, 72, 73, 184]	+/− [64, 73, 93, 96]	?	?	+ [64, 182]	?
Attention/memory	NMS	+ [64, 72, 73, 184]	+/− [64, 73, 93, 96]	?	?	+ [29, 36, 117]	?
Gastrointestinal functions	NMS	+ [72, 73]	+/− [64]	?	?	+ [182, 185] [29, 36, 117, 182, 185]	?
Urogenital functions	NMS	+ [72, 73, 76, 186]	+/− [73, 93, 96]	?	?	+ [29, 36, 117, 182, 185] [182, 185]	?
Sexual functions	NMS	− [72, 73]	+/− [73, 93, 96]	?	?	+ [29, 36, 117, 182, 185] [182, 185]	?
Miscellaneous	NMS	+ [64, 72]	+/− [64]	?	?	+ [29, 36, 117, 182, 185] [182, 185]	?

NMS reflect the categories of the Non-Motor Symptom Scale (NMSS), which is commonly used to assess non-motor symptoms in studies [187]

− No efficacy or deterioration in open or controlled; studies

? No studies or no positive expert consensus

+ improved according to expert opinion or open studies

++ improved according to controlled studies

*CSAI* continuous subcutaneous apomorphine infusion, *LCIG* levodopa–carbidopa intestinal gel, *LECIG* levodopa–entacapone–carbidopa intestinal gel, *CSFLI* continuous subcutaneous foslevodopa/foscarbidopa infusion, *DBS* deep brain stimulation, *MRgFUS* magnetic resonance-guided focused ultrasound, *MS* motor symptoms, *NMS* non-motor symptoms, *PDQ-39* Parkinson’s Disease Questionnaire-39, *PDQ-8* Parkinson’s Disease Questionnaire-8

***Motor symptoms****:* DBS has proven to be superior to other methods in the treatment of levodopa-refractory tremor, with STN- and GPi-DBS being similarly effective in suppressing tremors [36]. There are no controlled, randomized studies which compare DBS with pump therapies or comparing different pump therapies with each other [57, 64, 70, 73, 81, 94, 133, 134, 136, 137]. Overall, all assessed procedures notably improve MFs/dyskinesia, with LCIG and DBS demonstrating slightly stronger effects on Off-time duration compared to CSAI when considering weighted open-label studies [141].

More recently and formally after the appearance of the German guidelines, continuous subcutaneous foslevodopa/foscarbidopa infusion (CSFLI) therapy has been approved 2023 in Europe and initial assessments of its efficacy are available by now. CSFLI is a novel formulation of levodopa (LD) and carbidopa prodrugs, delivered via a continuous subcutaneous infusion. A phase 1 open-label, randomized, 2-period crossover study comparing the pharmacokinetics (PK) of CSFLI to LCIG demonstrated stable plasma concentrations with CSFLI [142]. The safety and efficacy of CSFLI were assessed in two key clinical trials. The first, a 12-week phase 3, randomized, double-blind trial, compared CSFLI with oral immediate-release levodopa–carbidopa (LD/CD) [132]. The results showed that CSFLI significantly increased “On” time without troublesome dyskinesia (mean difference 1.75 h) and reduced “Off” time (mean difference −1.79 h) compared to oral LD/CD. The CSFLI group also had higher rates of infusion-site adverse events (erythema, pain, cellulitis), but these were mostly non-serious and mild to moderate in severity. Despite a higher discontinuation rate (22% vs. 1% for oral LD/CD), the overall benefit–risk profile favored CSFLI. A second, 52-week open-label trial further evaluated the long-term safety and efficacy of CSFLI in patients with advanced PD [143]. Over 52 weeks, patients receiving CSFLI showed significant improvements in normalized “On” time without troublesome dyskinesia (mean change 3.8 h) and “Off” time (mean change −3.5 h). In addition, the percentage of patients experiencing morning akinesia decreased from 77.7% at baseline to 27.8% at week 52. Improvements in sleep quality and quality of life were also observed. Although infusion-site reactions remained the most common adverse event, the treatment was generally well-tolerated. Future studies will need to assess its long-term efficacy and comparative effectiveness with other advanced therapies.

***Non-motor symptoms:*** All procedures improve non-motor symptoms in general, though prospective non-randomized follow-up studies suggest minor differences among these symptoms. In the EuroInf 2 study, direct comparisons of LCIG, CSAI, and DBS showed significant improvements in various non-motor symptom domains. DBS effects on non-motor symptoms depend on electrode placement and stimulation parameters [144]. High-quality data also support procedure-specific effects on non-motor symptoms. For example, CSAI has been shown to improve motor symptoms, quality of life, sleep, mood, gastrointestinal, and urogenital symptoms, with notable improvements in sleep and quality of life [72, 145–149]. LCIG studies suggest efficacy in treating sleep disorders [85, 93, 150–152].

***Symptom constellations that may argue against an intervention*** (absolute/relative contraindications) guide decision-making, as some conditions may preclude intracerebral or intra-abdominal procedures. These may include severe brain atrophy (DBS) or severe diabetes with frequent infections (LCIG, LECIG). Interdisciplinary consensus is crucial in such cases, facilitated by interdisciplinary indication conferences.

***Age:*** Previously defined age limits for invasive therapies, particularly DBS, are no longer appropriate and should be supplanted by individual considerations of operability, therapy need, and expected outcomes.

***Cognition:*** Cognitive assessment is essential due to the common occurrence of cognitive disorders in PD patients. While certain neuropsychological instruments have been proposed, they lack robust data support. In principle, the prospects for motor improvement are not generally worse with cognitive impairment. Therapeutic decisions depend on the severity of the symptoms and the intervention [153]. Long-term data on DBS’s cognitive impact are mixed, with concerns over specific domains [154–156]. However, some studies report neutral or positive cognitive effects [22, 157, 158]. Overall, DBS should be avoided in patients with clear dementia according to recognized clinical criteria.

***Psychiatric diseases:*** Severe psychiatric disorders like depression or psychosis may exclude DBS candidacy [159]. In the case of hallucinations, it should be considered whether these are levodopa-induced or independent symptoms. Limited evidence suggests DBS may alleviate impulse control disorders (ICD) [58, 59, 160]. LCIG and CSAI also show promise in managing ICD [73, 161–163].

***Gait and balance disorders:*** The clinical efficacy of DBS hinges on levodopa responsiveness. Levodopa-independent gait and balance disorders cannot be treated with DBS or pump therapies [164, 165].

#### Combination of invasive therapies in the treatment of PD

In some cases, combining procedures may be necessary to treat recurring fluctuations or dyskinesia. Studies have demonstrated potential benefits, though cognitive decline and individual challenges necessitate careful consideration [166–170]. Data on specific combinations like LCIG and apomorphine or ablative procedures with DBS are sparse. A recent study (PMID: 37914414) suggests that in patients with PD, modifying or combining advanced treatments can improve motor function and subjective symptom reporting. This finding supports the potential benefits of combining treatment options, though further research is needed to confirm and refine this approach.

**Table:** 

Based on the available evidence, the following recommendations were agreed upon in the German PD Guideline: In principle, invasive procedures should particularly be considered if there are impairing levodopa-dependent fluctuations that cannot be sufficiently improved by optimizing oral/transdermal therapy The decision in favor of a particular procedure should consider not only the effectiveness on motor symptoms, but also non-motor symptoms and patient characteristics as well as the patient’s individual preference (Table 1), whereby factors should be weighted on a case-by-case basis and discussed in an interdisciplinary case conference together with the patient In the event of MF recurrence following an invasive procedure, the primary objective is to identify the underlying cause. In selected cases, a combination with a second invasive procedure may be considered The choice of follow-up procedure must be based on the individual patient profile at the time of the decision for a second procedure	
**Consensus strength:**	**100%, strong consensus**
